# Cardiac Autonomic Nervous System in Heart Failure: Imaging Technique and Clinical Implications

**DOI:** 10.2174/157340311795677725

**Published:** 2011-02

**Authors:** Mark J. Boogers, Caroline E. Veltman, Jeroen J. Bax

**Affiliations:** 1 1Department of Cardiology, Leiden University Medical Center, Leiden, The Netherlands; 2 The Interuniversity Cardiology Institute of The Netherlands, Utrecht, The Netherlands

**Keywords:** Cardiac 123-iodine metaiodobenzylguanidine imaging, heart failure, heart-to-mediastinum ratio, myocardial sympathetic innervation, prognosis, ventricular arrhythmia.

## Abstract

The autonomic nervous system interacts in the pathophysiology of heart failure. Dysfunction of the sympathetic nervous system has been identified as an important prognostic marker in patients with chronic heart failure. At present, cardiac sympathetic nerve imaging with 123-iodine metaiodobenzylguanidine [123-I MIBG] has been employed most frequently for the assessment of cardiac sympathetic innervation and activation pattern. The majority of studies have shown that cardiac sympathetic dysfunction as assessed with 123-I MIBG imaging is a powerful predictor for heart failure mortality and morbidity. Additionally, 123-I MIBG imaging can be used for prediction of potentially lethal ventricular tachyarrhythmias in heart failure patients. At present however, the lack of standardization of 123-I MIBG imaging procedures represents an evident issue. Standardized criteria on the use of 123-I MIBG imaging will further strengthen the clinical use of 123-I MIBG imaging in heart failure patients.

## CARDIAC AUTONOMIC NERVOUS SYSTEM IN HEART FAILURE

Chronic heart failure represents one of the most important challenges in clinical cardiology since several decades [1, 2]. The American Heart Association has recently updated their report on the heart disease and stroke statistics and indicated that approximately 5.3 millions patients are diagnosed with heart failure in the United States [2]. Additionally, heart failure was identified as the primary mode of death in 284,365 patients in the United States in 2004 [2]. Moreover, heart failure represents an important health care problem as the annual hospital admissions for heart failure increased considerably from approximately 400,000 in 1979 to more than 1,084,000 in 2005. As a result, an exponential increase has been observed in the direct and indirect costs involved in the diagnosis and treatment of heart failure, exceeding $34 billion in 2008 [2]. 

Heart failure is a pathophysiologic condition characterized by a reduced myocardial pump function which lacks the ability to supply peripheral tissues with sufficient metabolic requirements. Different pathophysiologic mechanisms have been related to the development and propagation of the clinical syndrome of heart failure, as shown in Table **1**. In case of a reduced cardiac function, several compensation pathways become activated that aim to preserve cardiovascular homeostasis. One of the these mechanisms that plays an essential role in patients with heart failure is the neurohormonalsystem, consisting of the adrenergic nervous system and the renin-angiotensin-aldosteron system  [RAAS] [3, 4]. In patients with heart failure, a decreased cardiac output results in an activation of the high-pressure baroreceptors of the left ventricle [LV], carotid sinus and aortic arch [4]. Activated baroreceptors generate afferent stimuli to cardio-regulatory centers of the central nervous system which leads to an activation of sympathetic nervous pathways. Additionally, a diminished effective renal blood flow  [caused by a reduced pump function] activates the RAAS system *via* the release of renin from the juxtaglomerular apparatus. Renin catalyzes the conversion of angiotensinogen  [produced in the liver] into the inactive angiotensin I. Subsequently, angiotensin I is metabolized into angiotensin II by the angiotensin converting enzyme, predominantly within the vascular bed of the lungs. Angiotensin II is involved in sodium- and water retention as well as vascular constriction. Furthermore, angiotensin II can lead to activation of other compensatory mechanisms in heart failure, including the sympathetic nervous system. Moreover, a reduced cardiac stroke volume results in an increased [LV] end-diastolic volume along with a rise in LV end-diastolic pressure. Based upon the Frank-Starling principle, these increased preload conditions will enhance the force of myocardial contraction, which compensates the decline in cardiac stroke volume.

In the early phase of heart failure, the hemodynamic consequences of a deteriorated cardiac pump function are compensated by the neurohormonal feedback mechanisms [3, 4]. However, in patients with chronic heart failure, the neurohormonal feedback mechanisms are detrimental as they may cause cardiac hypertrophy and fibrosis, leading to cardiovascular remodeling and restructuring. Additionally, myocardial β-adrenoceptors may have become desensitized and down-regulated in a chronic state of heart failure, leading to a progressive decline in cardiac function [3]. 

Accordingly, the sympathetic nervous system plays an essential role in the pathophysiology of the clinical syndrome of heart failure [3, 4]. Sympathetic stimuli are conducted *via* adrenergic fibers, located predominantly in the superficial subepicardium following the major epicardial coronary arteries [5]. In patients with a failing heart, the activated sympathetic nervous system can modulate heart rate  [chronotropic effect], atrioventricular conductance  [dromotropic effect] as well as the contractile force  [inotropic effect]. The noradrenergic function of the sympathetic nervous system is primarily mediated by the uptake and release of the neurotransmitter norepinephrine, which binds to adrenoceptors located on the specific target cells. Different mechanisms have been identified that modulate the uptake of norepinephrine from the presynaptic cleft, including the uptake-1  [neuronal] and uptake-2  [non-neuronal] mechanisms. The neuronal uptake  [uptake-1] of norepinephrine is performed by the norepinephrine transporter  [NET] protein located at the plasma membrane of the terminal dilated regions of the sympathetic neurone, whereas the extra-neuronal uptake  [uptake-2] is predominantly performed *via* the postsynaptic sodium-dependent, low-affinity transport mechanism. Currently, these pre- and postsynaptic uptake mechanisms have been used frequently to depict the sympathetic innervation and activation pattern in patients with chronic heart failure [3, 4, 6-7].

## SYMPATHETIC NERVE IMAGING

**Fig. (1):**
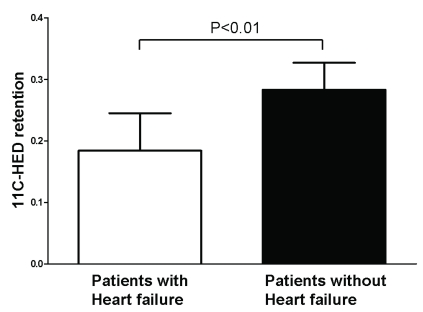
Cardiac sympathetic innervation can be assessed with positron emission tomography [PET] imaging with carbon-11 hydroxyephedrine [11C-HED]. Patients with heart failure showed significantly lower retention of 11C-HED when compared to patients without heart failure [0.18 ± 0.06 vs. 0.28 ± 0.04, p<0.01]. Data are based on reference 9.

To date, positron emission tomography  [PET] and single photon emission computed tomography  [SPECT] are the only two imaging modalities that permit visualization of sympathetic innervation and activation [6-7]. Moreover, both noninvasive imaging techniques allow assessment of global and regional abnormalities in myocardial sympathetic innervation. One of the major advantages of PET imaging remains the fact that it allows absolute quantification of abnormalities in sympathetic innervation with a superior spatial and temporal resolution as compared to SPECT imaging [7]. Additionally, it is important to note that the majority of PET tracers is more closely related to the endogenous neurotransmitters when compared to the tracers used for SPECT imaging. At present, hydroxyephedrine labeled with carbon-11  [11C-HED] represents the most commonly used PET tracer for sympathetic nerve imaging. The frequent use of 11C-HED for sympathetic nerve imaging may be explained by the fact that 11C-HED shows a high affinity for the NET protein  [uptake-1] located on the sympathetic neurone, and importantly, 11C-HED is not metabolized by catechol-O-methyl transferase  [COMT] or monoamine oxidase  [MAO] enzymes in the terminal endings of the sympathetic neurone. Thus far, different studies have sought to quantify global and regional abnormalities in sympathetic innervation in patients with heart failure using PET imaging [8-10]. Vesalainen *et al*. [8] performed an interesting study that evaluated myocardial retention of 11C-HED in patients with and without heart failure. The patient population consisted of 30 patients with mild to moderate heart failure, as reflected by New York Heart Association  [NYHA] functional class II/III, and 7 healthy control patients. Global cardiac retention index of 11C-HED was assessed in all patients using resting PET imaging. The study has shown that global 11C-HED retention index was significantly lower in patient with heart failure as compared to the healthy control patients  [0.29 ± 0.10 vs. 0.42 ± 0.07, p<0.01]. In addition, Pietilä *et al*. [9] evaluated the prognostic value of global cardiac 11C-HED retention in 46 patients with NYHA class II/III heart failure. During follow-up, cardiac mortality and heart transplantations were documented. The study has demonstrated that patients with heart failure showed significantly lower 11C-HED retention index when compared to patients without heart failure [0.18 ± 0.06 vs. 0.28 ± 0.04, p<0.01] (Fig. **1**). Moreover, Cox proportional hazards regression analysis showed that 11C-HED retention was significantly associated with cardiac mortality or the occurrence of heart transplantation  [p=0.014]. Additionally, regional abnormalities in sympathetic nerve innervation have been assessed with PET imaging and 11C-HED, as shown in Fig. (**2**) [9, 10]. Hartmann and colleagues [10] have evaluated sympathetic innervation pattern in patients with dilated cardiomyopathy and moderate-to-severe heart failure. In total, 29 heart failure patients and 8 healthy control patients underwent dynamic PET imaging to assess regional abnormalities in 11C-HED retention using the 9-segment model, placed over the polar map. Tracer activity was expressed per cardiac segment as an absolute or relative  [percentage of the maximal tracer activity] percentage. Patients with heart failure showed a significantly lower retention of 11C-HED than patients without heart failure  [6.2 (1.6) %/min vs. 10.7 (1.0) %/min, p<0.01]. Importantly, as compared to the healthy controls, considerable reduction in 11C-HED retention was observed in the apical  [p<0.01] and inferoapical  [p<0.05] segments in patients with dilated cardiomyopathy and heart failure. Despite the potentials of PET imaging, several factors have been acknowledged that hamper its large-scale clinical use, including the complex production and delivery of short-live PET tracers, extensive processing requirements and limited availability of PET cameras. Furthermore, its widespread use has been restricted by the fact that PET imaging is a costly imaging technique [the production of PET tracers as well as PET equipment are expensive] when compared to other non-invasive imaging techniques [7].

**Fig. (5):**
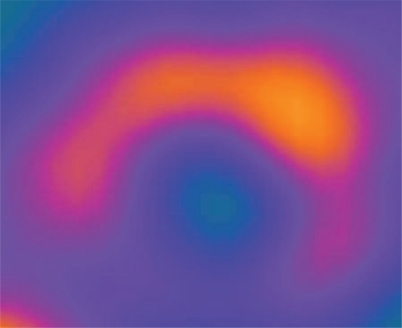
Regional abnormalities in cardiac sympathetic innervation can be assessed with 123-iodine metaiodobenzylguanidine [123-I MIBG] single photon emission computed tomography [SPECT] imaging. This is an example of a patient with a marked defect in the sympathetic innervation of the inferior wall.

Currently, 123-iodine metaiodobenzylguanidine  [123-I MIBG] represents the most commonly used radiopharmaceutical agent for evaluation of the sympathetic innervation pattern in patients with heart failure, originating from ischemic or non-ischemic cause [11-15]. MIBG is a false neurotransmitter that uses the same uptake and storage mechanisms as the endogenously produced neurotransmitter norepinephrine. Different mechanisms have been identified that facilitate the uptake of MIBG from the synaptic cleft, including the uptake-1  [neuronal] and uptake-2  [non-neuronal] mechanisms. In human hearts, the neuronal uptake of MIBG plays the most important role, since MIBG uptake was absent in denervated myocardium of patients with previous cardiac transplantation [16]. Importantly, MIBG represents an interesting and potent radiopharmaceutical agent for sympathetic nerve imaging as it has been shown to provide strong signal intensity due to the fact that MIBG is accumulated in storage vesicles of the sympathetic neurone, without further metabolic degradation. The signals are used for planar and SPECT imaging which are usually performed at two different intervals; early planar and SPECT images are acquired 15 minutes after tracer administration, whereas delayed planar and SPECT images are acquired 4 hours after tracer administration, as illustrated in Fig. (**3**)  [17]. From planar images, semiquantitative measurements of 123-I MIBG uptake can be derived that provide information on global innervation pattern, as shown in Fig. (**4**). Currently, the most commonly used semi-quantitative parameter of 123-I MIBG uptake is the heart-to-mediastinum  [H/M] ratio which is calculated by dividing the mean counts per pixel within the cardiac region of interest by the mean counts per pixel within the upper mediastinum. In addition, 123-I MIBG SPECT imaging provides information on regional sympathetic innervation of the myocardium (Fig. **5**).

## PROGNOSTIC VALUE OF 123-I MIBG IMAGING IN PATIENTS WITH HEART FAILURE

Over the recent decades, a large number of studies have sought to determine the prognostic value of sympathetic nerve imaging with 123-I MIBG in patients diagnosed with chronic heart failure [11-15, 18]. The 123-I MIBG studies have shown that patients with heart failure show a reduced myocardial uptake of 123-I MIBG, and more importantly, these 123-I MIBG studies have indicated that heart failure patients with the lowest myocardial 123-I MIBG uptake tend to have the worst prognosis [11-15, 18]. In this perspective, an interesting study was performed by Merlet *et al*. [13] who sought to determine whether 123-I MIBG imaging could predict clinical outcome in patients with heart failure. In total, 112 patients with idiopathic dilated cardiomyopathy and NYHA II-IV heart failure underwent sympathetic nerve imaging with 123-I MIBG. Additionally, patients underwent M-mode echocardiography, chest X-ray, right-sided heart catheterization, and exercise testing to identify potential predictors for clinical outcome. Furthermore, the plasma norepinephrine concentration was determined in all patients. Over a mean follow-up of 27 ± 20 months, cardiac mortality was documented in 27 patients and cardiac transplantation in 19 patients. Multivariate stepwise regression analysis revealed that MIBG uptake  [p<0.01] and circulating norepinephrine  [p<0.01] were independently associated with cardiac mortality during follow-up. Furthermore, Cohen-Solal *et al*. [18] evaluated the hypothesis that reduced sympathetic innervation, as assessed with 123-I MIBG imaging, was associated with poor outcome in heart failure patients. The prospective analysis was based on 93 patients with chronic heart failure  [either due to ischemic or dilated cardiomyopathy] and depressed LV systolic function [LV ejection fraction  [LVEF] <45%]. Late H/M ratio  [p=0.04] and oxygen consumption  [VO2]  [p<0.01] were significantly associated with all-cause mortality or the occurrence of cardiac transplantation. Importantly, patients with a late H/M ratio <1.27 showed a significantly lower survival rate as compared to patients with a late H/M ratio >1.27  [p<0.01].

Beyond myocardial 123-I MIBG uptake, planar imaging can also be used to assess the myocardial washout rate in patients with heart failure [19-22]. Myocardial washout rate has been recognized as an indicator of the sympathetic cardiac tone, as it reflects the degree in which MIBG is washed out of the myocardium over time. So far, a large number of studies have evaluated the predictive value of myocardial washout rate, and have shown that heart failure patients with an increased cardiac washout rate are at risk for adverse cardiovascular events [19-21]. An important study was performed by Ogita *et al*. [20] who evaluated whether sympathetic nerve imaging with 123-I MIBG was able to predict clinical outcome in 79 patients with chronic heart failure. The patient population was divided into patients with a low  [<27%] or high  [>27%] myocardial washout rate. The cutoff value was derived from the mean value of the myocardial washout rate from the patient population. In total, 23  [29.1%] patients showed an adverse cardiac event over a mean follow-up of 31 months. Additionally, morbidity  [p<0.01] and mortality  [p<0.01] rates were significantly higher in patients with increased cardiac washout rate as compared to patients without an increased cardiac washout rate. Another important study was performed by Yamada *et al*. [21] who compared the prognostic value of 123-I MIBG parameters with heart rate variability  [HRV] in 65 patients with mild-to-moderate heart failure and reduced LV systolic function  [LVEF <40%]. Patients were followed-up from study enrollment to cardiac death or first documented hospitalization for worsening heart failure. Multivariate Cox proportional hazards regression analysis showed that myocardial washout rate was independently associated with the occurrence of cardiac death or hospitalization [HR 1.072, 95% CI 1.01-1.14, p=0.029]. Accordingly, these studies have shown that myocardial uptake  [H/M ratio] and washout  [washout rate] of 123-I MIBG can be used for prediction of outcome in patients with heart failure.

**Fig. (6):**
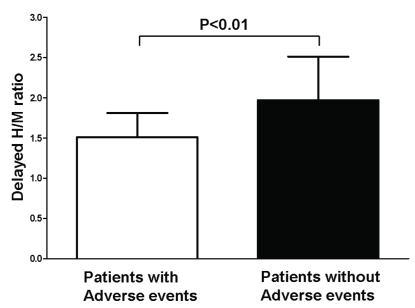
Differences in heart-to-mediastinum [H/M] ratio in patients with and without adverse events. Delayed H/M ratio was significantly lower in heart failure patients with adverse events when compared to patients without adverse events during follow-up [1.51 ± 0.30 vs. 1.97 ± 0.54, p<0.01]. Data based on reference 14.

In addition to single-center studies, Agostini *et al*. [14] performed a retrospective multicenter study evaluating the predictive value of 123-I MIBG variables derived from previously acquired 123-I MIBG images. More specifically, the multicenter study was designed to evaluate whether a standardized quantitative approach yielded similar results when compared to the previously reported findings from single-center studies. In total, 290 patients with NYHA functional class II-IV heart failure and depressed LV systolic function  [LVEF <50%] were enrolled. All 123-I MIBG scans were analyzed in one core laboratory by three experienced and independent observers. The delayed H/M ratios were obtained from planar imaging using a standardized approach. The late H/M ratios were related to the occurrence of major cardiovascular events during follow-up, including documented cardiac death, heart transplantation and potentially lethal ventricular arrhythmia. The study has shown that patients with major cardiovascular events showed significantly lower late H/M ratio as compared to patients without adverse cardiovascular events  [1.51 ± 0.30 vs. 1.97 ± 0.54, p<0.01] (Fig. **6**). Additionally, multivariate analysis revealed that late H/M ratio and LVEF were independently associated with the occurrence of adverse events. Accordingly, the study has shown that late H/M ratio could be used for identification of heart failure patients at risk for adverse events. Importantly, the study has demonstrated that the use of a standard quantitative approach yielded similar results when compared to previously findings from single-center studies, regardless of differences in imaging protocols and technical equipment.

## 123-I MIBG IMAGING FOR IDENTIFICATION OF PATIENTS AT RISK FOR VENTRICULAR ARRHYTHMIAS

Abnormalities of the autonomic nervous system, as assessed with 123-I MIBG imaging, is also thought to interact in the pathophysiology of ventricular tachyarrhythmias [23, 24]. It has been postulated that viable myocardium with deprived sympathetic innervation may show an exaggerated and hypersensitive response to circulating catecholamines. More specifically, an increased automaticity and enhanced triggering may be observed in denervated viable myocardium [23]. Pilot studies have related reduced myocardial uptake of 123-I MIBG with the occurrence of ventricular arrhythmias [25, 26]. Arora* et al*. [27] performed a pilot study evaluating the hypothesis that sympathetic nerve imaging with 123-I MIBG could be used for prediction of appropriate implantable cardioverter-defibrillator  [ICD] therapy. Appropriate ICD therapy  [defined as the first documented ICD therapy in response to ventricular tachycardia or ventricular fibrillation] was used as a surrogate for potentially lethal ventricular tachyarrhythmias. For comparison reasons, the patient population was divided into patients with  [n=10] or without  [n=7] appropriate ICD therapy during follow-up. Patients with appropriate ICD therapy showed significantly reduced global and regional 123-I MIBG uptake as compared to patients without appropriate ICD therapy during follow-up  [p<0.05]. The authors have postulated that 123-I MIBG imaging can be used for the identification of patients at risk for potentially lethal ventricular tachyarrhythmias. Another important study was performed by Tamaki *et al*. [22] who evaluated whether 123-I MIBG imaging could be valuable for prediction of sudden arrhythmic death in 106 consecutive patients diagnosed with chronic heart failure. The primary study endpoint was defined as the occurrence of sudden arrhythmic death, whereas secondary endpoints were defined as cardiac death or death due to progressive heart failure. In total, 38  [36%] patients died over a mean follow-up of 65 ± 31 months, including 18  [17%] patients who died because of sudden cardiac death. In multivariate analysis, myocardial washout rate [HR 1.052, 95% CI 1.020-1.085, p<0.01] and LVEF [HR 0.930, 95% CI 0.870-0.995, p=0.0341] were independently associated with sudden arrhythmic death. Importantly, both parameters were significant predictors for cardiac death or death due to progressive heart failure.

The value of 123-I MIBG SPECT imaging for prediction of ventricular arrhythmias has also been demonstrated in several studies [26, 28]. Recently, Bax *et al*. [26] evaluated whether 123-I MIBG imaging was associated with inducibility of ventricular arrhythmias during electrophysiologic testing. In total, 50 patients underwent electrophysiologic testing, 123-I MIBG and technetium-99m tetrofosmin imaging. Among all variables, only the summed defect score on delayed 123-I MIBG SPECT imaging was significantly different between patients with or without a positive electrophysiologic test. Patients with a positive electrophysiologic test showed significantly larger 123-I MIBG SPECT defects as compared to patients without a positive electrophysiologic test [42.7 ± 8.8 vs. 34.9 ± 9.8, p<0.05]. Furthermore, the hypothesis that 123-I MIBG imaging may be useful for prediction of potentially lethal ventricular arrhythmias was recently evaluated in 116 patients currently indicated for ICD therapy [28]. Before ICD implantation, all patients underwent myocardial perfusion imaging to assess myocardial infarction and perfusion abnormalities. Additionally, patients underwent sympathetic nerve imaging with 123-I MIBG prior to ICD implantation. During follow-up, appropriate ICD therapy  [used as a surrogate of potentially lethal ventricular tachyarrhythmias] and cardiac mortality were documented. In this study, the primary endpoint was defined as the first documented appropriate ICD therapy, whereas the composite of appropriate ICD therapy and cardiac death was defined as the secondary endpoint. Over a mean follow-up of 23 ± 15 months, appropriate ICD therapy was reported in 24  [21%] patients and cardiac death in 13  [11%] patients. Importantly, 123-I MIBG SPECT defect score on delayed imaging was an independent predictor for both endpoints. Moreover, patients with extensive sympathetic denervation  [defined as summed late 123-I MIBG SPECT defect score>26] showed significantly more appropriate ICD therapy  [52% vs. 5%, p<0.01] when compared to patients with limited sympathetic denervation  [summed late 123-I MIBG SPECT defect score ≤26] at 3-year follow-up. Accordingly, 123-I MIBG planar and SPECT imaging can be used for the identification of patients at risk for potentially lethal ventricular tachyarrhythmias.

## IMPLEMENTATION OF 123-I MIBG IMAGING INTO CLINICAL PRACTICE

Accordingly, abnormalities in cardiac sympathetic innervation or activation play an important role in prognostication of patients with heart failure. A detailed evaluation of abnormalities in cardiac sympathetic nervous system can be performed by cardiac 123-I MIBG imaging. Cardiac 123-I MIBG images can be acquired safely in nearly all patients with heart failure, except in patients with known hypersensitivity for MIBG compounds or MIBG sulphate. Despite the potentials of 123-I MIBG imaging, some impeding factors have been recognized that hamper the clinical implementation of sympathetic nerve imaging with 123-I MIBG [29]. One of the important factors that has been identified is the lack of standardization and validation of cardiac 123-I MIBG imaging and post-processing procedures [29, 30]. At present, 123-I MIBG studies have shown considerable heterogeneity in the use of technical equipment, imaging protocols and quantitative post-processing analysis [11-13, 15, 29]. A large number of studies has used low-energy parallel-hole collimators for 123-I MIBG image acquisition, whereas some studies have used medium-energy parallel-hole collimators [31-35]. It has been demonstrated that the type of collimator used for 123-I MIBG imaging plays an evident role in the assessment of H/M ratios; scatter artifacts due to penetration of the collimator septa by high-energy photons are less prominent with the use of medium-energy collimators when compared to low-energy collimators [34-36]. Moreover, studies have demonstrated that the use of medium-energy collimators  [with thicker septa] results in superior accuracy of semi-quantitative assessment of myocardial 123-I MIBG uptake [34-36]. Additionally, the semi-quantitative assessment of myocardial 123-I MIBG uptake and washout can be calculated using different approaches. In the study by Cohen-Solal *et al*. [18], who sought to determine the prognostic value of cardiac 123-I MIBG imaging in 93 heart failure patients, the myocardial washout rate was calculated using the following formula:  [(H early – H delayed) X 100] / H early. In contrast, the myocardial washout rate was calculated with another approach in the study by Ogita *et al*. [20]; the myocardial washout rate was calculated by the following equation:  [(H early – M early) – (H late – M late)] / (H early – M early). Additionally, differences in myocardial 123-I MIBG uptake and washout rate can be related to the fact that some studies did not correct for isotope decay [20]. Accordingly, improved standardization of 123-I MIBG imaging and post-processing procedures is currently indicated. Recently, Flotats* et al*. [17] have published a proposal for standardization of 123-I MIBG imaging, including a detailed description of patient preparation, data acquisition and post-processing of 123-I MIBG images. Development of such standardizations on the use of 123-I MIBG imaging may further strengthen the clinical value of sympathetic nerve imaging with 123-I MIBG in patients with heart failure.

## POTENTIAL TECHNICAL PITFALLS OF 123-I MIBG IMAGING

Although cardiac 123-I MIBG imaging can be used for risk stratification of patients with heart failure, some potential pitfalls of cardiac 123-I MIBG imaging should be addressed. First, the evaluation of 123-I MIBG SPECT images may be hampered by severely reduced myocardial uptake of 123-I MIBG in patients with advanced heart failure. Furthermore, the readers should be aware of the fact that 123-I MIBG shows a reduced uptake in the inferior wall under normal conditions [37]. Particularly, elderly patients may show reduced uptake of 123-I MIBG in the inferior wall. Additionally, 123-I MIBG is also taken up by peripheral tissues that are supplied by sympathetic nerves, including the liver, lungs and thyroid gland [17]. Moreover, 123-I MIBG can be present within the gastrointestinal system of many patients. In patients with excessive extra-cardiac 123-I MIBG uptake, evaluation of 123-I MIBG SPECT images  [particularly evaluation of the inferior wall] may be difficult. In patients with non-diagnostic 123-I MIBG scans, plasma norepinephrine levels or the rate of norepinephrine spillover can be used for evaluation of sympathetic activation.

## CONCLUSION

The autonomic nervous system plays an essential role in the pathophysiology of heart failure. Dysfunction of the sympathetic nervous system represents an important prognostic marker in patients with chronic heart failure. Most commonly, 123-I MIBG imaging has been used for the assessment of abnormalities in cardiac sympathetic innervation and activation. Cardiac sympathetic denervation as assessed with 123-I MIBG represents an important predictor for heart failure mortality and morbidity. Furthermore, 123-I MIBG imaging can be used for prediction of potentially lethal ventricular tachyarrhythmias in patients with heart failure. At present however, improved standardization of 123-I MIBG imaging procedures is indicated. Standardized criteria on the use of 123-I MIBG imaging may further strengthen the clinical value of 123-I MIBG imaging in heart failure patients.

## Figures and Tables

**Fig. (2):**
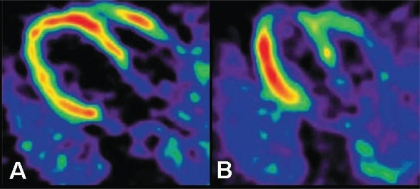
Positron emission tomography [PET] imaging with hydroxyephedrine labeled with carbon-11 [11C-HED] allows assessment of cardiac sympathetic innervation. Moreover, PET imaging with 11C-HED can be used for assessment of regional abnormalities in cardiac sympathetic innervation in patients with heart failure, originating from either dilated [panel A] or ischemic [panel B] cardiomyopathy. Reprinted with permission from reference 9.

**Fig. (3):**
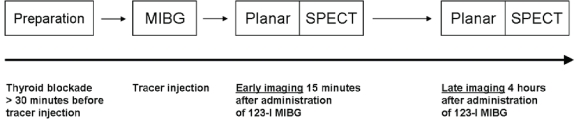
Schematic illustration of the cardiac 123-iodine metaiodobenzylguanidine [123-I MIBG] imaging protocol. First, thyroid blockade is usually performed with oral administration of potassium perchlorate or iodine at least 30 minutes prior to 123-I MIBG acquisition. Once 123-I MIBG has been administered, cardiac images are acquired according to a two-step protocol consisting of planar and single photon emission computed tomography [SPECT] imaging performed at 15 minutes [early imaging] and 4 hours [late imaging].

**Fig. (4):**
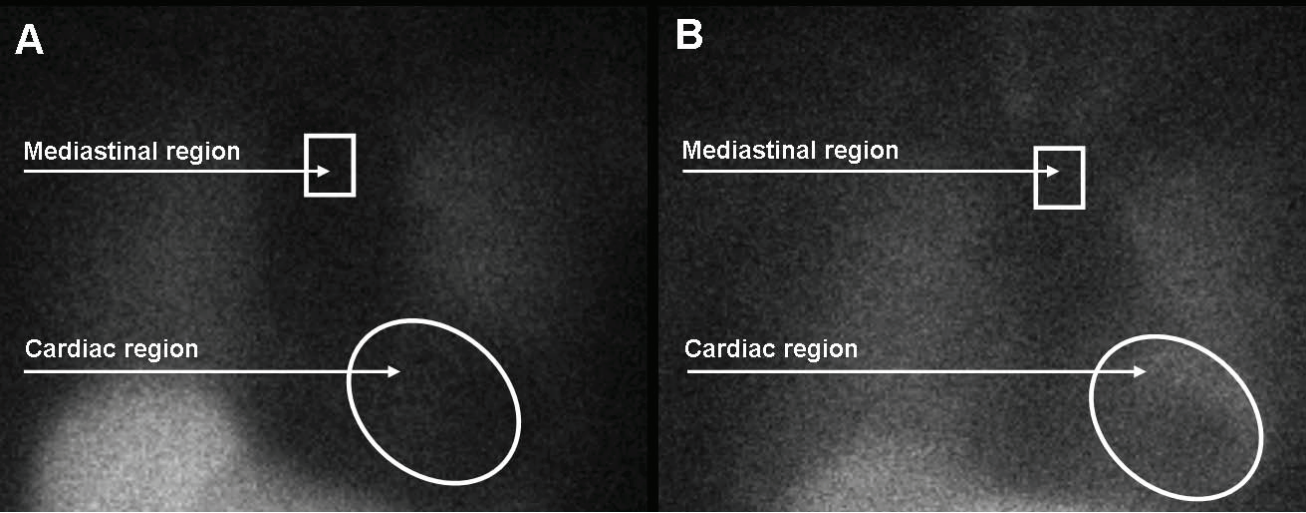
Global sympathetic innervation can be assessed from planar imaging with 123-iodine metaiodobenzylguanidine [123-I MIBG]. The heart-to-mediastinum [H/M] ratio is calculated by dividing the mean counts per pixel within the cardiac region of interest by the mean counts per pixel within the upper mediastinum. **A**. Example of a patient with heart failure and reduced global uptake of 123-I MIBG within the myocardium. The late H/M ratio was 1.31. **B**. Example of a patient with heart failure and normal global uptake of 123-I MIBG within the myocardium. The H/M ratio was 1.80 on delayed planar imaging.

**Table 1:** Overview of Common Causes of Chronic Heart Failure

**Myocardial disorder** Coronary artery disease Hypertrophic cardiomyopathy Hypo / hyperthyroidism Neurohormonal excess Inflammatory or immune Metabolic disorder or infiltrative Toxic Idiopathic cardiomyopathy **Hemodynamic overload** Hypertension Renal failure **Cardiac arrhythmia** Atrial / ventricular arrhythmias Sinus node dysfunction Atrioventricular conduction block **Cardiac valve disease** Mitral valve stenosis or regurgitation Aortic valve stenosis or regurgitation **Pericardial disease** Constrictive **Extra-cardiac pathology** Anemia Sepsis Iatrogenic **Congenital heart disease** Congenitally corrected transposition of great arteries Univentricular heart or double inlet left ventricle Tetralogy of Fallot Pulmonary atresia Ventricular and atrial septal defect

## ABBREVIATIONS

11C-HED: = Carbon-11 hydroxyephedrine labeled
ICD: = Implantable cardioverter-defibrillator
123-I MIBG: = 123-iodine metaiodobenzylguanidine
LV: = Left ventricle / left ventricular
LVEF: = Left ventricular ejection fraction
NET: = Norepinephrine transporter
NYHA: = New York Heart Association
PET: = Positron emission tomography
RAAS: = Renin angiotensin aldosteron system
SPECT: = Single photon emission computed tomography
